# Proposing a conceptual framework for integrated local public health policy, applied to childhood obesity - the behavior change ball

**DOI:** 10.1186/1748-5908-8-46

**Published:** 2013-04-18

**Authors:** Anna-Marie Hendriks, Maria WJ Jansen, Jessica S Gubbels, Nanne K De Vries, Theo Paulussen, Stef PJ Kremers

**Affiliations:** 1 Academic Collaborative Centre for Public Health Limburg, Regional Public Health Service, Geleen, The Netherlands; 2 Department of Health Promotion, Caphri, School of Public Health and Primary Care, Maastricht University, Maastricht, The Netherlands; 3 Department of Health Services Research, Caphri, School of Public Health and Primary Care, Maastricht University, Maastricht, The Netherlands; 4 Department of Health Promotion, NUTRIM, School for Nutrition, Toxicology and Metabolism, Maastricht University, Maastricht, The Netherlands; 5 TNO (Netherlands Organisation of Applied Scientific Research) Healthy Living, Leiden, The Netherlands

**Keywords:** Conceptual framework, Intersectoral collaboration, Integrated approach, Health policy, Childhood obesity, Prevention, Behavior change, Organizational change, Local government

## Abstract

**Background:**

Childhood obesity is a ‘wicked’ public health problem that is best tackled by an integrated approach, which is enabled by integrated public health policies. The development and implementation of such policies have in practice proven to be difficult, however, and studying why this is the case requires a tool that may assist local policy-makers and those assisting them. A comprehensive framework that can help to identify options for improvement and to systematically develop solutions may be used to support local policy-makers.

**Discussion:**

We propose the ‘Behavior Change Ball’ as a tool to study the development and implementation of integrated public health policies within local government. Based on the tenets of the ‘Behavior Change Wheel’ by Michie and colleagues (2011), the proposed conceptual framework distinguishes organizational behaviors of local policy-makers at the strategic, tactical and operational levels, as well as the determinants (motivation, capability, opportunity) required for these behaviors, and interventions and policy categories that can influence them. To illustrate the difficulty of achieving sustained integrated approaches, we use the metaphor of a ball in our framework: the mountainous landscapes surrounding the ball reflect the system’s resistance to change (by making it difficult for the ball to roll). We apply this framework to the problem of childhood obesity prevention. The added value provided by the framework lies in its comprehensiveness, theoretical basis, diagnostic and heuristic nature and face validity.

**Summary:**

Since integrated public health policies have not been widely developed and implemented in practice, organizational behaviors relevant to the development of these policies remain to be investigated. A conceptual framework that can assist in systematically studying the policy process may facilitate this. Our Behavior Change Ball adds significant value to existing public health policy frameworks by incorporating multiple theoretical perspectives, specifying a set of organizational behaviors and linking the analysis of these behaviors to interventions and policies. We would encourage examination by others of our framework as a tool to explain and guide the development of integrated policies for the prevention of wicked public health problems.

## Background

This article addresses key questions that arise within the context of integrated public health policies (*e.g.*, ‘Healthy Public Policy’ [1] or ‘Health in All Policies’ [2-4]) and introduces a conceptual framework to study and guide their development. In most countries, such policies are developed by local policy-makers who work within local governments (*i.e.*, municipal authorities) [3-12], so we focus on policy development at local government level. We focus on policies that aim to prevent ‘wicked’ public health problems [13,14] (*e.g.*, childhood obesity [15]), since such problems defy traditional intra-sectoral problem-solving approaches and therefore require innovative integrated approaches in which health and non-health sectors collaborate (*i.e.*, intersectoral collaboration) [16-19].

Despite differences between countries or between the states of federal countries in the involvement of national or provincial governments, the roles, functions, and types of governance structures [20], and in policy approaches to public health problems (*e.g.*, smoking or gun control), the core of policy development for wicked public health issues remains similar in most countries [21-26]. In the Netherlands, for example, the national government sets priorities every four years that are then operationalized (*i.e.*, developed into a health policy document) by local policy-makers [9-12], while in the United States, most policy priorities are set by state (rather than national) government and then operationalized by local policy-makers [25,26]. The core of public health policy-making with respect to wicked problems remains the need to implement an integrated approach aimed at collaboration between different (health and non-health) sectors. Assisting local policy-makers, public health professionals and researchers in developing and implementing integrated public health policies requires a conceptual framework to study and guide this development and implementation effort [17,18], so our goal was to develop such a framework.

**Figure 1:**
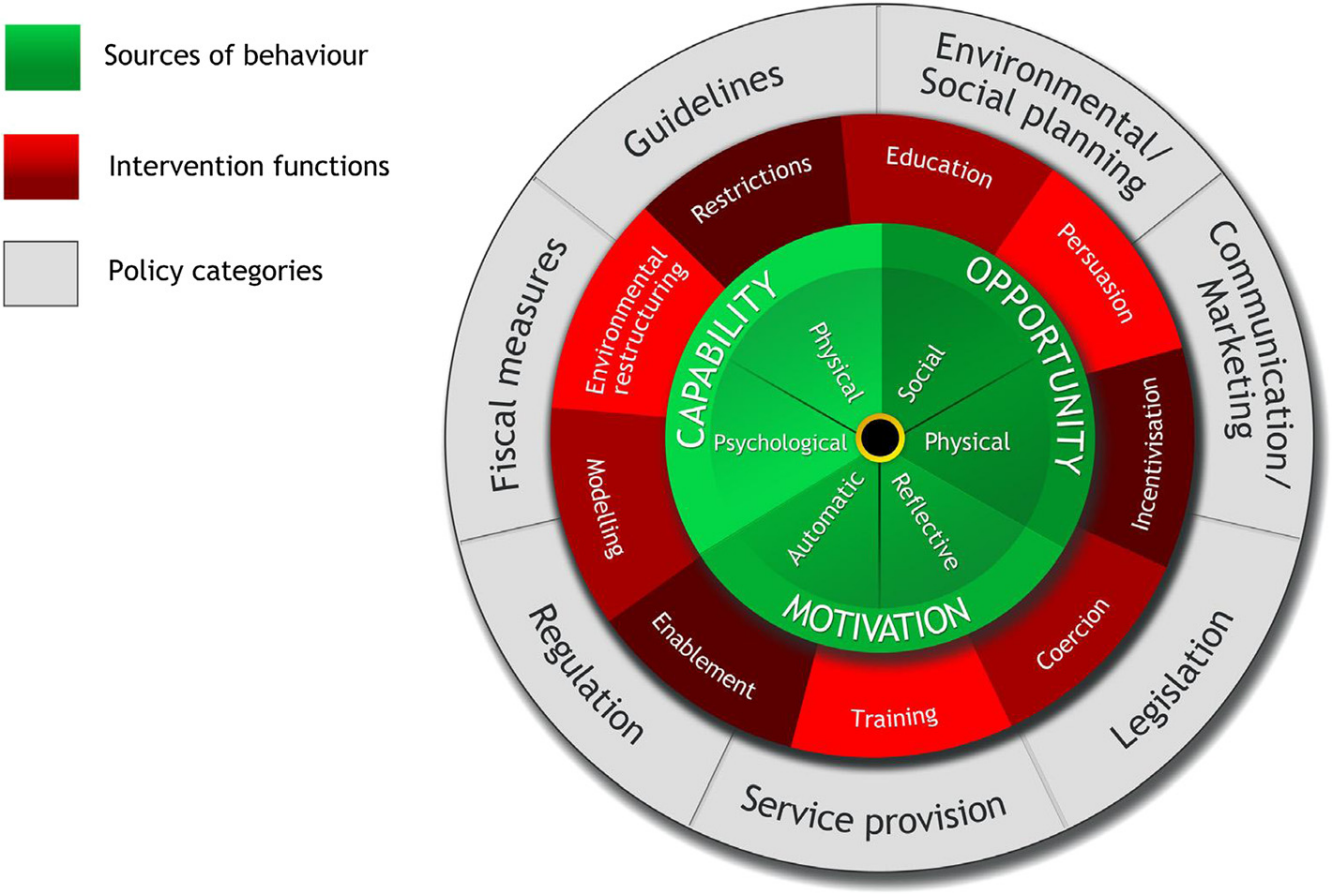
The behavior change wheel.

Our framework was mainly inspired by the ‘Behavior Change Wheel’ (BCW) (Figure 1) that was recently presented by Michie and colleagues [27]. Since the BCW was developed from an extensive review of existing frameworks and has been tested in other theoretical domains (primary implementation) [27,28], it provided a sound basis for the development of our own framework. We extended the BCW so it could be used as: a practical tool to assist local policy-makers and those who support them in overcoming barriers to developing and implementing integrated public health policies to prevent wicked public health problems; and as a theoretical tool to drive empirical research and stimulate theory development in the field of local integrated public health policies to prevent wicked public health problems.

The development of our framework was guided by the research question: ‘How are integrated public health policies for the prevention of wicked public health problems developed?’ Data were collected among policy actors and categorized into ten organizational behaviors expected to be at the core of policy development.

The aims of this paper are: to reflect on the complexity of making integrated public health policy for wicked public health problems; to reflect on the context in which integrated public health policies for wicked public health problems are developed; and to introduce a framework for the development and implementation of integrated public health policies. To illustrate the Behavior Change Ball (BCB), we use the development of public health policies for childhood obesity prevention at the local government level as an example throughout this article.

## Why focus on childhood obesity prevention through integrated public health policies?

Prevalence rates of childhood obesity have doubled over the last three decades, and approximately 170 million children (< 18 years) worldwide are now estimated to be overweight or obese [29-32]. Childhood and adolescent obesity is associated with poorer subjective as well as objective health [33-40] and often tracks into adulthood [41]. Consequently, it causes huge rises in healthcare costs, affecting economic growth [42,43]. In view of these consequences, governments are increasingly focusing their attention on preventing childhood obesity *e.g.*, [44,45]. However, though much research is available on the determinants of childhood obesity, it has not yet been clearly established how this information can be used to develop effective prevention approaches [32]. The complex interactions between so-called ‘thrifty genes’ [46], consumerist life-styles and ‘obesogenic’ urban environments [47], make it difficult to decide on the right avenue for prevention [48].

Experts argue that significant health improvements can be achieved by focusing on factors at all levels of society within and outside the health sector [1-4,49-57]; they therefore recommend that governments implement so-called ‘integrated approaches’ (including integrated policies) that are characterized by a combination of coordinated interventions by multiple organizations and sectors, and are developed through intersectoral collaboration, (*i.e.*, ‘collaboration between the most relevant sectors within and outside the health domain to improve public health’ [18]). Examples of policies developed through intersectoral collaboration are preventing the establishment of fast food restaurants near schools and increasing the safety of playgrounds in deprived neighborhoods. Such policies can only be implemented if zoning policies (policies that regulate the size, type, structure and use of land or buildings in designated areas) developed by the department of spatial planning are aligned with the goals of the public health department (see [58] for 93 examples of such policies). Since such integrated policies have the potential to decrease the availability of energy-dense cheap foods and increase children’s physical activity levels [59], their development is of great interest to those who seek to prevent wicked public health problems such as childhood obesity [32,60,61]. In practice, however, a wide range of content- and process-related factors (Table 1) [62-90] appear to hamper the development and implementation of integrated public health policies for such wicked public health problems [91-94].

**Table 1:** Barriers regarding development and implementation of integrated public health policies, as reported in the literature

***Content-related barriers***	**Reference**
Lack of awareness of the childhood obesity problem in non-health sectors.	Aarts *et al. *[62]
The Dutch Law on Public Health has decentralized the public health tasks to local governments. With regard to jurisdiction, the public health policy domain has a position similar to other jurisdictions such as public safety. In practice, however, public health is not a dominant policy domain: resources for public health are limited, and other jurisdictions (*e.g.*, public safety) are considered important issues, while health promotion is considered less interesting, depending on the political priority given to certain policy domains.	Law on Public Health [9]
	Breeman *et al. *[63]
	Steenbakkers [64]
‘Wicked’ nature of obesity makes it very unattractive to invest in its prevention.	Head [14]
	Head and Alford [19]
Decreasing the incidence of childhood obesity is very unlikely within the short timeframe in which most politicians work (determined by election frequencies).	Head [14]
	Aarts *et al. *[62]
	Romon *et al. *[65]
	Blakely *et al. *[66]
Difficulty of developing consensus about ways to tackle the problem due to the lack of hard scientific evidence about effective solutions.	Han *et al. *[25]
	Aarts *et al. *[62]
	Head [14]
	Trivedi *et al. *[67]
	National Institute for Health and Clinical Evidence [68]
Framing of childhood obesity (especially by neo-liberal governments) as an individual health problem instead of a societal problem. Responsibility for achieving healthy-weight promoting lifestyles is thus shifted completely away from governments to individual children and their parents.	Hunter [69]
	Dorfman and Wallack [70]
	Schwartz and Puhl [71]
Lack of political support.	Aarts *et al. *[62]
Ambiguous political climate: governments do not seem eager to implement restrictive or legislative policy measures since this would mean they have to confront powerful lobbies by private companies.	Nestle [72]
	Peeler *et al.*[73]
Lack of presence of champions and political commitment	Verduin *et al. *[74]
	Woulfe *et al. *[75]
	Bovill [76]
***Process-related barriers***	
Local government officials lacking the knowledge and skills to collaborate with actors outside their own department.	Steenbakkers [64]
Insufficient resources (time, budget).	Aarts *et al. *[62]
	Steenbakkers [64]
	Woulfe *et al. *[75]
Lack of membership diversity in the collaborative partnerships, resulting in difficulties of implementation	Woulfe *et al. *[75]
Lack of clarity about the notion of intersectoral collaboration.	Harting *et al. *[17]
Not being clear about the aims and added value of the intersectoral approach.	Bovill [76]
Top-down bureaucracy and hierarchy, disciplinarity and territoriality, sectoral budgets, and different priorities and procedures in each sector.	Bovill [76]
Inadequate organizational structures.	Steenbakkers [64]
	Woulfe *et al. *[75]
	Alter and Hage [77]
	Hunter [33]
	Warner and Gould [2]
Poor quality of interpersonal or interorganizational relationships.	Woulfe *et al. *[75]
	Isett and Provan [78]
Top management not supporting intersectoral collaboration.	Bovill [76]
Lack of involvement by managers in collaborative efforts.	Steenbakkers *et al. *[79]
Lack of common vision and leadership.	Woulfe *et al. *[75]
	Hunter [62]
Innovation in local governance is hampered by:	Borins [80]
- asymmetric incentives that punish unsuccessful innovations much more severely than they reward successful ones	
- absence of venture capital to seed creative problem solving	
- disincentives lead to adverse selection: innovative people choose careers outside the public sector.	
Adaptive management – flexibility of management required, focusing on learning by doing.	Head and Alford [19]
	Holling [81]
Lack of communication and insufficient joint planning.	Axelsson and Axelsson [82]
Hierarchical governance instead of network governance	Warner and Gould [2]
Barriers are related to the ‘niche’ character of the sectors involved:	Jansen [83]
Achieving the unique advantage of collaboration, which is referred to as ‘synergy,’ is harder in diverse groups, but at the same time such diverse groups have the potential to lead to greater synergy compared to collaboration within homogeneous groups.	Jansen *et al.*[84]
	Jones [85]
	Lasker and Weiss [86]
	Miller and
	Watson and Johnson [87]
	Hendriks *et al.*[88]
	Hoffman *et al.*[89]
	Paulus [90]
Implementation not being considered a dominant part of the planning and policy process	Bovill [76]

## Which theories can explain integrated policy development, and what are their limitations?

A wide range of theories can be used to explain the development of integrated public health policies [17,18]. Some theories describe a continuum of integration *e.g.*, [95-98], while others focus on intersectoral, cross-sectoral or multisectoral collaborations, coalitions and partnerships *e.g.*, [99-103]. In addition, there are theories with a broader focus, which can also be applied to understanding intersectoral collaboration, such as individual behavior change theories *e.g.*, [104], diffusion and implementation theories [105,106], and organizational change theories *e.g.*, [107]. Other theories describe processes of policy-making: coalition theories [108,109] focus on the role of policy subsystems, while technocratic [110,111], garbage-can [112,113], and incremental models [114] describe how policies are developed.

Each of the above theories offers unique and useful insights, but they have three important limitations, making it difficult to apply them satisfactorily to the local government setting. First of all, most of these theories apply only to specific aspects of collaboration, and together do not provide a comprehensive approach. Kingdon’s stream theory [112], for example, is very useful for the conceptualization of agenda setting, which is an important part of the policy-making process, but it is not able to account for other parts of the policy process (*e.g.*, implementing policy solutions). Although such theories are very useful for fundamental research (in which the creation of immediately useful knowledge is not the primary purpose) [115-117], action-oriented researchers and especially the policy-makers themselves need ‘actionable knowledge’ [118], *i.e.*, knowledge that can guide the way to solutions after barriers or facilitators within the process have been identified.

A second limitation is that most of the theories are based on research within organizational settings rather than within governmental settings. Although we recognize that local governments are also organizations, the conditions in non-governmental organizations are very different from those in local governments, so research results derived from non-governmental settings cannot be directly transferred to that of local government [119,120]. For example, local policy-makers have to work within a context: of policies that are delegated to them by national governments; of a democratic political system leading to changes in government policies after every new parliamentary election, making it difficult to work towards long-term goals; in which mistakes made by the authorities are highlighted in the media since citizens are critical about the way governments spend their tax money, so tolerance of errors is low; with a far more hierarchical organizational structure than that of a typical non-governmental organization; and in which policy implementation is often not under their own control or in their own interest, while in non-governmental organizations, policies are usually implemented by the same organization that has developed them [76,119].

A third limitation of theories to explain the development of integrated public health policies is that most policy-making models are developed for simple or fairly uncomplicated public health problems (*i.e.*, tame problems) [19,110,121]; such policy models fail to take into account the factors that make policy development for complex public health problems (*i.e.*, wicked problems) difficult (Table 1) [19,110]. Current policy models usually distinguish among several policy-making stages, such as problem definition, selecting policy solutions, gaining political and public support for the policy solution, policy implementation, evaluation of the policy, and dissemination of effective policies [12,110]. These stages represent the practice of policy formulation when clear policy goals can be established, adequate information is available, and appropriate methods can be chosen that can lead to activities that efficiently and effectively achieve these goals. However, these preconditions are violated when policies for the prevention of wicked public health problems are developed. Since neither the problem nor the solution is perceived in the same way by the many different parties involved [19], current policy-making models cannot be satisfactorily used to explain the development of policies for such problems within local governments.

To overcome these limitations, we developed a more comprehensive conceptual framework. Although some researchers have argued that it is unlikely that a single comprehensive framework can be developed [17], progress in this field can only be made if researchers are willing to invest effort in developing such a framework.

## Which theories provided the basis of our current framework?

We used two conceptual models as the basis of our framework. Following Jansen [83], we distinguished categories of local policy-makers (*e.g.*, strategic, tactical and operational levels), and we also adopted the core concepts of the BCW (capability, opportunity, motivation, and behavior, or ‘COM-B’; intervention functions, policy categories, and the relationships between them). In addition, we integrated theories from political and policy science, organizational science, marketing, psychology, and health science [95-114] to achieve a cross-fertilization that might lead to new insights.

## Extensions to the behavior change wheel

Our main inspiration was the Behavior Change Wheel (BCW) by Michie *et al.*[27] (Figure 1). This framework was developed from an extensive review of existing frameworks, and has been tested in other theoretical domains (primary implementation) [28]. The function of the BCW is to link an analysis of target behavior (the ‘B’ from the COM-B model of behavior) to intervention functions and policies. When we tried to apply it to our target population, *i.e.*, local policy-makers, however, we encountered a limitation of the BCW with regard to our context. In our context, local policy-makers, public health professionals and researchers would first need to define which organizational behaviors need to be introduced, reinforced or replaced for the development and implementation of integrated public health policies. We considered that pre-defining a set of organizational behaviors based on theories might support the users of the framework. The current framework thus builds on the principles of the BCW, but modifies the ‘behavioral goals’ by specifying relevant organizational behaviors and linking them to policy-makers at the strategic, tactical and operational levels.

We wanted to provide a theoretical framework that could function: as a practical tool to assist local policy-makers and those supporting them in overcoming barriers to developing and implementing integrated public health policies to prevent wicked public health problems; and as a theoretical tool to drive empirical research and stimulate theory development in the field of local integrated public health policies to prevent wicked public health problems. We therefore decided to extend the BCW in three ways, which are outlined below.

## Extension 1: different target population

In contrast to Michie *et al.*[27], who applied the BCW to the behaviors of the traditional target population of health-promoting interventions (*i.e.*, intermediaries and the ultimate target group of people who are assisted in a health behavior change process), we had a target population consisting of the ‘enablers’ of health promotion interventions, namely local policy-makers*.* Furthermore, since our target population is tied to the organization in which they work (the local government) we decided to refer to their behavior as ‘organizational behavior’ rather than just ‘behavior’ [122]. These organizational behaviors may consist of collective and individual behaviors and can also be seen as critical factors or processes for the development and implementation of integrated public health policies.

## Extension 2: adding a second function

**Figure 2:**
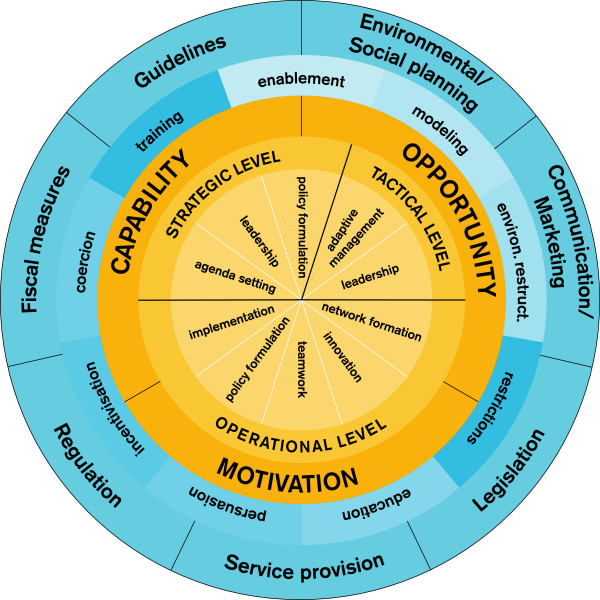
**The behavior change ball, adapted from Michie *et al.*’s **[27]**behavior change wheel.** The yellow parts of the framework depict the diagnostic function of the framework: an assessment of the policy context in which integrated public health policies should be developed and implemented. The blue parts depict the heuristic function of the framework: based on the diagnosis, the framework guides the way to solutions (interventions and policies). Compared to the Behavior Change Wheel, the Behavior Change Ball also specifies organizational behaviors and relates them to the most relevant actors, categorized into three hierarchical levels that can be found in local governments; these are displayed as ‘wedges’ (agenda setting, leadership, policy formulation, adaptive management, network formation, innovation, teamwork, policy formulation, and implementation) and levels (operational, tactical, strategic). In the Behavior Change Wheel, the ‘wedges’ are not specified, but are displayed as a black dot at the center, which reflects a single specific behavioral goal [27]. Our specification of the behavioral goals into ten wedges adds a second function to the Behavior Change Wheel, making our framework more comprehensive, which is what we needed to explain and guide the development and implementation of integrated public health policies.

By adding organizational behaviors that are indicative of an integrated approach, the ‘hub of the wheel’ becomes not only a heuristic tool (linking an analysis of behavior to theory-based interventions and policies) but also a diagnostic tool within the context of local government. Thus, the original goal of the BCW (heuristic) has been extended by a second function: providing a structure to categorize the most important aspects of an integrated approach (*i.e.*, functioning as a diagnostic tool), as depicted in the yellow parts of the model (Figure 2). To include such a diagnostic function, it was necessary to pre-define a set of organizational behaviors that will enable an assessment of the current situation in local government organizations with regard to the development and implementation of integrated public health policies.

## Extension 3: adding a third dimension

**Figure 3:**
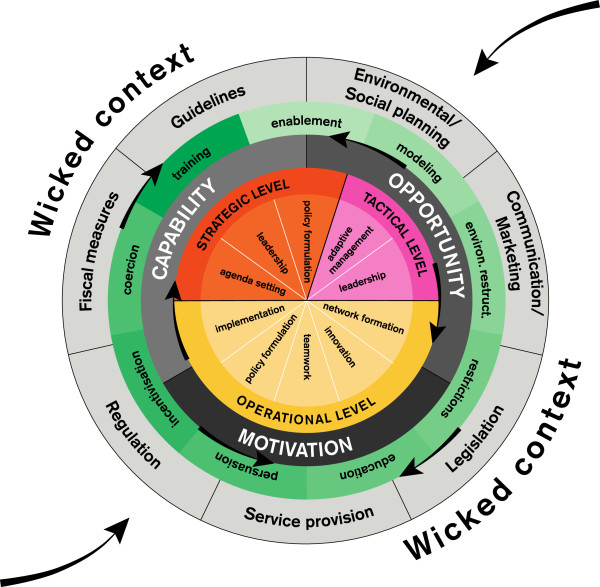
**All circles can rotate independently.** The Behavior Change Ball consists of circles that reflect organizational behaviors, actors within three hierarchical levels, determinants of organizational behaviors, interventions, and policies or programs. Policies or programs enable interventions, and determinants are necessary for each of the organizational behaviors that are related to actors at the operational, tactical, or strategic level.

**Figure 4:**
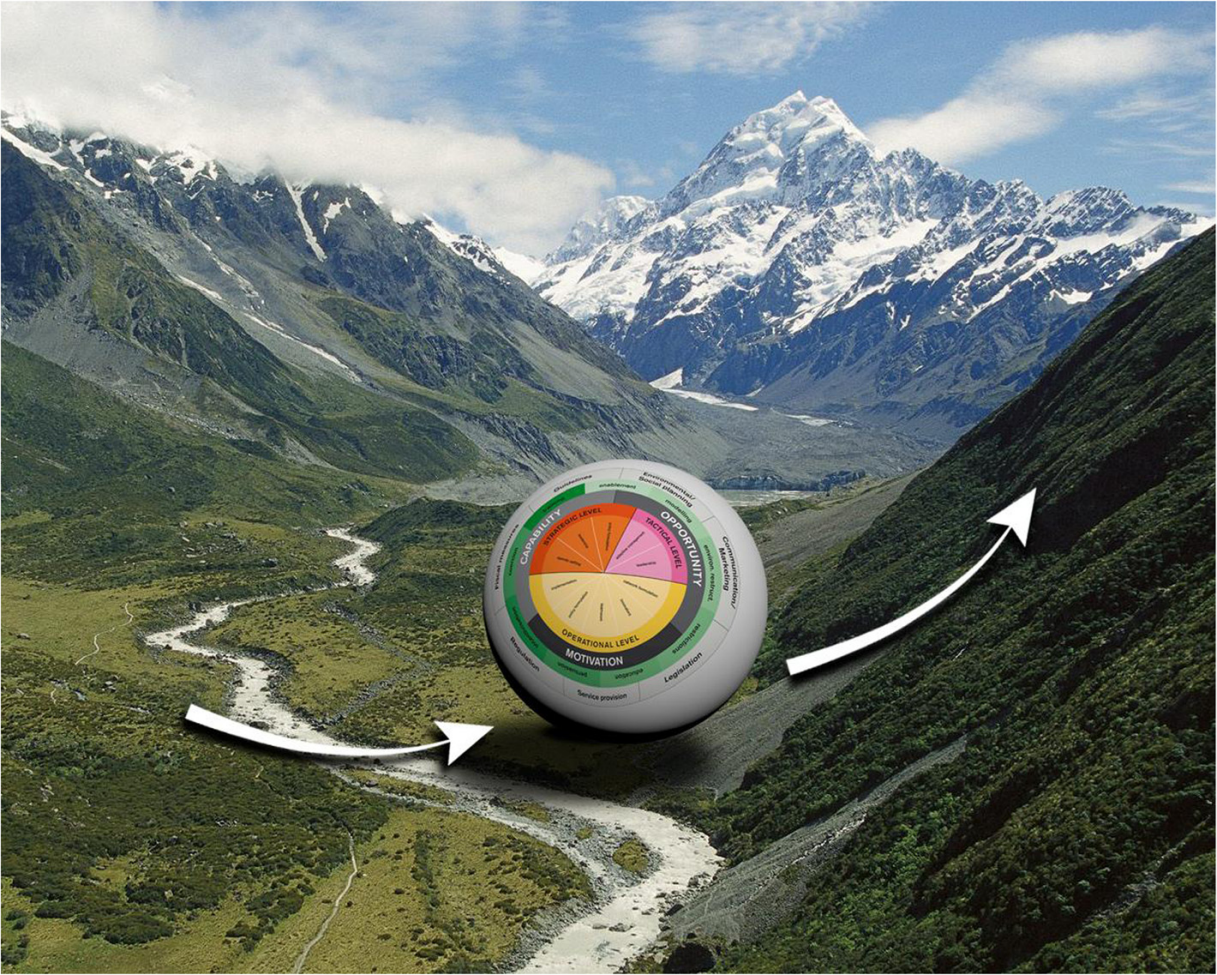
**The landscape and the behavior change ball.** The proposed relationships between the theoretical concepts from the Behavior Change Ball are best illustrated by the metaphor of a ball moving through a landscape.

Since each of the concepts in our framework can strengthen the initiation, implementation and continuation of effective policies, the dynamics of the political and obesity-related environmental context prompted us to use the metaphor of a ball that is rolling around in a mountainous landscape (Figures 3 and 4). This metaphor could explain why current implementation attempts have often failed. The steep hills surrounding the ball reflect the systems’ resistance to change; the forces of gravity make it difficult to roll a ball towards a mountain peak. Therefore, we decided to ‘reinvent the wheel’ (which is two-dimensional) and develop it into a ball (three-dimensional). The metaphor of a ball moving through a landscape has also been applied successfully in other research areas to reflect the dynamics that are at work in complex systems [123,124]. In the following sections, we present our proposed framework, the ‘Behavior Change Ball,’ with which we aim to enhance empirical research grounded in theory.

## The behavior change ball

Before outlining the components of the ‘Behavior Change Ball’ (BCB) (COM-B, intervention functions, and policy categories) and its application, we describe its development and target group.

## How was the framework developed?

To identify the ten organizational behaviors (displayed in the wedges) that need to be carried out by certain levels of local policy-makers, we interviewed local policy-makers at strategic, tactical, and operational levels within several Dutch local governments, attended meetings of the public health service in one Dutch region, developed theoretical reflections [95-114] and held discussions with experts in the field of integrated public health policy development, politics, and intersectoral collaboration. We related the organizational behaviors to the organizational levels at which the behavior needed to be carried out. For example, we found that agenda setting is controlled by local policy-makers at the strategic level (*e.g.*, the municipal executive), while local policy-makers at the tactical level (*e.g.*, heads of departments) are responsible for adaptive management. By categorizing these organizational behaviors, we aimed to integrate them into one comprehensive framework. After having designed an early version of this framework, we discussed it with experts and key informants. Based on their recommendations, we adapted the framework where necessary. To increase the generalizability of our framework, to improve the construct definition of its concepts, and to raise the data to a theoretical level, we constantly compared our data with relevant literature and similar or alternative frameworks. The outcome of this inductive and iterative research process was our conceptual framework (Figures 2, 3, 4) [125].

## Who are the target group of the framework?

The target group of our framework consists of the local policy-makers who are involved in developing integrated public health policies. Local policy-makers work within a complex environment in which members of the municipal executive and local politicians (strategic level) direct local government managers (tactical level) and professionals (operational level) towards the development and implementation of certain policies. They can be divided into three levels reflecting the kind of decisions they make [64,83]. Simply stated, policy-makers at the strategic level (the municipal executive, known in the Netherlands as the College of Mayor and Aldermen) decide ‘what will be done within the organization,’ while tactical level policy-makers (heads of departments) decide ‘how (and sometimes also when) it will be done’ (*e.g.*, which preconditions have to be fulfilled), and operational level policy-makers (civil servants) decide ‘who will do what and when’ (*e.g.*, how to achieve a goal). These levels are related to the traditional levels of top management, middle management, and operational management [126], or Mintzberg’s strategic apex, middle line, and operational core [127]. To develop integrated public health policies, these three levels should collaborate vertically (between levels) as well as horizontally (between the sectors within one level) [64,83].

Despite attempts to involve the ultimate (*e.g.*, children and their parents) and intermediate (*e.g.*, commercial organizations within the community) target populations of health-promoting interventions in the process of developing policies, they are at a greater distance from the policy-making process than the local policy-makers themselves [128]. Therefore, we do not regard them as our key target group, but as external influences; they include, for example, international ambassadors for childhood obesity prevention (like Michelle Obama), experts advising the local policy-makers, and other levels of government (national, state, provincial, international).

## Which organizational behaviors encourage integrated health policies to be developed?

Ten wedges, displayed at the hub of the ball, represent a categorization of ten organizational behaviors (*e.g.*, agenda setting) that are relevant to the development of integrated public health policies. We decided to categorize the organizational behaviors to a specific level of local policy-makers. For example, agenda setting is formally the responsibility of the municipal executive and therefore categorized under the strategic level in our proposed framework. Although we acknowledge that others can influence agenda setting (*e.g.*, by reminding the executive to think about childhood obesity prevention), they are not officially in charge of it, and are therefore considered external influences situated in other parts of our proposed framework (*e.g.*, as determinants or interventions). Each of the organizational behaviors is discussed in more detail below.

## Organizational behavior 1: agenda setting at the strategic level

A new policy can only be developed if a problem attracts enough attention to appear on the political agenda [112]. Agenda setting is defined as: ‘the first stage of the public policy process during which some issues are given attention by policymakers and others receive minimal attention or are neglected completely’ [129]. In the case of childhood obesity prevention by means of integrated approaches, many agendas need to be set. Compared to mono-sectoral approaches, a much wider and more diverse group has to develop a shared vision, agree upon a strategy, and decide to invest resources [52,129]. Only then do lower level, local policy-makers (the tactical and operational levels) feel facilitated to elaborate on this shared vision [130]. A policy entrepreneur can stimulate agenda setting [112,131]. For example, a local alderman might visit a neighboring municipality and inspire them to give priority to childhood obesity prevention.

## Organizational behavior 2: leadership at the strategic level

Leadership can be defined as: ‘a process of social influence’ [132]. Such influence is especially important for the prevention of public health problems, since the benefits of prevention only become visible in the long run [32,52]. Prevention is therefore not of great interest to most politicians, who tend to work with shorter time frames [62,65,66,69,93]. To overcome this lack of interest, leaders in the effort to improve public health should be politically aware and skilled in formulating a clear, integrated vision of the public health problem: when defining the problem of childhood obesity, leaders need to emphasize the wicked nature of obesity and guide the search for systemic solutions [69,132,133]. For example, an alderman can emphasize that we all have created an ‘obesogenic environment’ and that overweight children and their parents are not solely to blame.

## Organizational behavior 3: policy formulation at the strategic level

Formulating policies is the *raison d’être* of governments; it is the process of translating agenda topics into a set of policy measures [134]. Policies can be formulated at the various governmental levels, each with its own goals [135]. This section focuses on policies at the strategic level. Strategic level policies set out the vision and strategy for a problem, guide the debate and set out the tasks for local policy-makers at the lower levels of the municipal hierarchy (the tactical and operational levels). They are symbolic and aim to motivate people or create momentum, and can give agenda topics a permanent character by securing resources [112,135]. For example, the program proposed by the municipal executive might include a section securing the resources that will be invested in implementing the integrated approach towards overweight prevention.

## Organizational behavior 4: adaptive management at the tactical level

The management of wicked public health problems requires an adaptive approach, which is characterized by an emphasis on learning from available evidence and utilizing this evidence in experiments [81,136,137]. This is especially important when addressing problems such as obesity, since most ‘solutions’ for childhood obesity prevention are not yet firmly rooted in scientific evidence. Adaptive management is an instrument that is used to change and learn about the system [81]. This implies that heads of departments, who are the day-to-day managers of the officials working in local government, should adopt an open and learning attitude and involve people such as researchers to evaluate their policies. Such an attitude stimulates the creativity of local policy-makers at the operational level, which is needed for the development of innovative policies [107,130]. For example, when managers are skeptical toward new working methods, such as intersectoral collaboration, new experiences will not be created, and officials will not learn new collaboration skills.

## Organizational behavior 5: leadership at the tactical level

Leadership at the tactical level is important since the integrated approach to childhood obesity prevention requires that policies are developed in a new way, *viz.* through intersectoral rather than intra-sectoral collaboration. Officials from different policy sectors (*e.g.*, spatial planning and public health) should have the opportunity to jointly lead the process of development and change [138]. Leadership by the heads of the departments is expected to be very important to facilitate this change process; they should support their subordinates in producing innovations [130]. For example, a manager might create new performance indicators that also incentivize officials who have successfully implemented initiatives for the integrated approach; this can create a culture in which others might also want to collaborate.

## Organizational behavior 6: network formation at the operational level

A network is defined as: ‘a group of interdependent but autonomous actors that come together to produce a collective output (tangible or intangible) that no one actor could produce on its own’ [77]. Networks that also involve non-health sectors should be formed to implement the integrated approach [2-4]. To attract these non-health stakeholders, actors from the health sectors should move out of their ‘comfort zone’ and ‘niche’ [83,133]. For example, spatial planning officials should be involved in the implementation of certain policies that require changes to physical environments. Additionally, such networks can boost agenda setting by mobilizing actors and increasing the collective capacity to confront opponents [1,112].

## Organizational behavior 7: innovation at the operational level

Innovation is currently very important within the policy context, since the traditional ways of solving childhood obesity problems have failed [139]. Innovators are the gatekeepers for the introduction of new ideas into the network. They are defined as: ‘the first individuals to adopt an innovation’ [106]. Innovation is becoming increasingly important in the policy process since national governments are encouraging local governments to implement integrated policies through public-private partnerships. In attempts to achieve such changes, an innovator may be the key to the exchange of new ideas between public and private organizations and may bridge the gaps between them. For example, officials might be motivated to use their contacts with the local supermarket to implement some of their policy ideas, but might need to overcome resistance from others within the organization who are afraid that the risk of failure of such collaboration is too high.

## Organizational behavior 8: teamwork at the operational level

Based on the initiatives developed by the network, the core of the network should take further initiatives through ‘teamwork’: ‘a set of interrelated thoughts, actions, and feelings of each team member that are needed to function as a team and that combine to facilitate coordinated, adaptive performance and task objectives resulting in value-added outcomes’ [140]. Currently, actors in the public health services are not yet fully accustomed to working in teams that include local policy-makers from different policy sectors, and thus are confronted by a totally new way of working. To be able to capitalize on their knowledge and skills [141], they need other ‘new’ competencies and tools [64,142-144], for example, spanning boundaries between problems and solutions, and bringing diverse partners together.

## Organizational behavior 9: policy formulation at the operational level

Teamwork by local policy-makers and other relevant stakeholders results in decisions being made on the way strategic policies are translated into operational policies (*i.e.*, ‘work’ or ‘action’ plans). They are action-oriented instead of symbolic. In contrast to strategic policies, operational policies translate the policy goals into concrete actions ready for implementation. Operational planning documents should describe the policy goals, instruments, and actions in a specific, measurable, acceptable, realistic, and time-bound (SMART) format [135], for example by describing when a law will be implemented that bans vending machines from primary schools.

## Organizational behavior 10: implementation at the operational level

Policies can only impact childhood obesity rates when they are implemented properly, so it is very important that implementation is considered a part of the planning and policy process [76]. Although this seems like stating the obvious, governments are usually judged on their policy documents rather than on the implementation of their policies [76,145]. Lack of implementation is therefore a commonly cited problem in the governmental context [76,119]. Usually, a package of policy measures is developed by policy-makers, but in the subsequent implementation stage, most of the measures need to be implemented by other actors than the local policy-makers themselves [119]. It is therefore important to involve outside stakeholders in policy development at an early stage [74,105,106,145] and to regularly evaluate implementation efforts to tackle current implementation obstacles and anticipate potential barriers for continuation [105,106,145].

## What determinants need to be present to achieve a particular organizational behavior?

The second circle of the model displays three categories of interrelated determinants of behavior: capability, opportunity, and motivation (COM) [27] (Figures 1–3). These determinants are needed for each of the ten organizational behaviors to occur. Other research fields such as marketing *e.g.*, [146], health sciences *e.g.*, [147], policy science *e.g.*, [112], and implementation science *e.g.*, [28] also use this categorization. Capability, opportunity, motivation, and behavior (‘COM-B’) are united in a ‘behavioral system’; if determinants are insufficiently present, COM-B may not function appropriately, and the behavior may not be established.

## How are the determinants conceptualized?

‘Capability’ is the extent to which individuals can adapt to change, generate new knowledge, and continue to improve their performance [148]: ‘capability is what people are able to do and to be’ [149]. ‘Psychological capability’ refers to the ability to engage in the necessary thought processes, such as comprehension and reasoning [27], and is closely related to competence, which refers to what individuals know or are able to do [148]. Important aspects in the context of intersectoral collaboration are assumed to be boundary-spanning, collaboration, and leadership skills [69,150]. There is also a ‘physical capability,’ but this is not directly relevant to this paper [27].

‘Opportunity’ refers to conditions that are external to the individual actor [27], that is, all social, political and organizational resources within a specified system that interact with the local policy-makers [105,106]. Two forms of opportunity are distinguished: physical and social. Physical opportunity is afforded by the environment (*e.g.*, organizational structures). Social opportunity refers to the milieu that dictates the way that we think about things, the words and concepts we use, and the predominant discourse (*e.g.*, organizational culture) [27].

‘Motivation’ can be divided into reflective and automatic processes. Reflective motivation involves reflective decision-making processes involving analytical choices or intentions (*e.g.*, evaluation and plans) [27]. An example is deciding to collaborate with other sectors since one has positive beliefs about intersectoral collaboration. Automatic motivation involves processes in which emotions and impulses that arise from associative learning or innate dispositions lead to certain choices [27]. Examples of automatic motivation are resistance to change or work engagement [142,151-153].

## Which interventions can influence the COM-B?

If the COM-B is suboptimal, interventions might be needed to increase the likelihood that certain organizational behaviors are effectively accounted for [27,154]. They are outlined below.

‘Education’ involves increasing knowledge and understanding [27]. Since policy sectors are not always aware of the way their policies influence health [62], education might increase awareness among all policy sectors and stimulate intersectoral collaboration. An example of a tool to create such awareness is Health Impact Assessment [154,155].

‘Persuasion’ means that communication is used to elicit or enhance positive or negative feelings or to stimulate action [27]. A national politician could, for example, persuade local, economically oriented politicians that obesity prevention is worth investing in because of the economic consequences of obesity in terms of work absenteeism in the future [154].

‘Incentivization’ means that expectations of rewards are created [27]. Incentivization is based on marketing and learning theory principles of direct reinforcement [146,156]. Reward systems that are built into the organizational structure, such as bonuses, are an example of incentivization since they can stimulate individuals by offering financial rewards [154].

‘Coercion’ means the use of punishment or costs [27], for example to force municipalities to subject their policies to a Health Impact Assessment [154,155].

‘Training’ can be used to overcome skills-related problems [27]. For example, attracting the right stakeholders for the development of integrated policies requires negotiation skills that might be trained [154].

‘Restriction’ refers to rules defining which behaviors are allowed or not allowed [27]. Institutions incorporate not only formal rules but also informal rules that shape the behavior of those working in them and thus may hamper intersectoral collaboration [157]. For example, performance management can restrict collaboration, especially when tight budgets result in a tendency to return to ‘core business’ [154].

‘Environmental restructuring’ is intended to change the social or physical context [27]. Changes in the social context refer to changes in culture (*e.g.*, pressure from the media), while changes in the physical context refer to changes in the structure (*e.g.*, institutional arrangements) [154,157,158]. A good example is the work of celebrity chef Jamie Oliver: media attention has enabled him to put the poor quality of school lunches on the political agenda.

‘Modeling’ provides an example that people can and like to copy [27]. It is based on social learning theories [156]. Managers may act as a model for the type of collaboration they want to encourage across policy sectors [64,79,154], and well-known mayors like Michael Bloomberg [159] may act as models to invest in local obesity prevention through policies [154].

‘Enablement’ means creating new ways to deal with or remove barriers [27]. At the strategic level, for example, a barrier to intersectoral collaboration, *viz*. ‘not having a shared goal,’ might be removed by an official having two policy sectors, such as spatial planning and public health, in their portfolio [154].

## Which policies can enable the interventions?

Nine policies are displayed in the outermost circle. They enable particular interventions and are outlined below [27].

‘Communication and marketing’ involves using print, electronic media, telephone or broadcast media [27]. For example, in order to achieve broad political commitment for the prevention of childhood obesity, a local alderman might be appointed as ambassador.

‘Guidelines’ involve documents that recommend or mandate practice [27]. An example might be using a contract to formalize network activities to make sure that commitments for investing in childhood obesity prevention are followed up.

‘Fiscal measures’ involve the use of the tax system to reduce or increase the financial cost of certain activities that might affect childhood obesity, for example by subsidizing municipalities that develop and implement integrated public health policies. Such financial support can stimulate local governments to invest in intersectoral collaboration, since innovating current working practices often requires additional investment of resources [105,106].

‘Regulation’ involves establishing rules or principles of behavior or practice [27]. Pooling resources, for example, can be seen as a working principle that fosters intersectoral collaboration; when targets are set for the governmental system as a whole, officials from the economic or spatial planning departments can share resources with health sectors and therefore become direct stakeholders of public health.

‘Legislation’ involves making or changing laws [27]. Laws aim to change behavior in a non-voluntary manner [146]. An example is the Dutch law on public health; the Dutch national government obliges local governments to produce a health policy document every four years [9], and the Health Care Inspectorate checks whether the laws are adhered to [160].

‘Environmental or social planning’ involves designing and/or controlling the physical or social environment [27]. An example is giving attention to the design of the organizational structure so it does not obstruct intersectoral collaboration.

‘Service provision’ involves delivering services [27]. Examples include offering specific training courses for civil servants who want to use social marketing to prevent obesity, or training courses on how to select evidence-based interventions.

## How can the behavior change ball be applied?

The framework can be applied within local governments by local policy-makers or those who assist them (*e.g.*, action-oriented researchers) to develop and implement integrated public health policy for the prevention of wicked public health problems. It can be applied for practical or theoretical purposes.

For practical purposes, the following four steps should be taken. First, identify the local policy-makers’ organizational behaviors that are described in the wedges of the ball (*i.e.*, not assessing the COM-B from scratch as in the BCW); this assessment should identify which organizational behaviors need to be introduced, reinforced, or replaced. For example, it may become clear that childhood obesity prevention is not on the agenda of the aldermen who is responsible for it (agenda setting). Second, based on the identification of the organizational behaviors that need attention, an analysis of the COM-B needs to indicate what might be an important avenue for improvement. For example, to set the agenda, the aldermen might first need to be informed about the severity of childhood obesity (agenda setting through increasing motivation). Michie *et al. *[27] describe how to select appropriate interventions (third) and policies or programs (fourth) to change the COM-B. For example, communication (policy) enables modeling (intervention) and influences automatic motivation, which may lead to agenda setting (organizational behavior) at the strategic level (our target population).

For theoretical purposes and to enable further study, the BCB can be used to structure or map data. For example, the BCB’s constructs can be used as topic lists or coding systems, or to map data from observations, interviews, or policy documents. Applying the BCB may reveal the value of certain theories in explaining the development and implementation of integrated public health policies and thus provide directions for further research.

## What are the limitations of this study?

A limitation of this study is that the linkages it identifies between the organizational behaviors are based on one research study. Although we grounded the linkages in existing theoretical assumptions and literature, we acknowledge that they should be further tested. We therefore hope to inspire other researchers to conduct more theory-based empirical research to validate and refine the BCB. Another limitation of this study could be that this framework was developed in the Netherlands, and may thus not be valid for countries where local governments bear less responsibility for developing public health policies. Also, our categorization of local government actors might appear less appropriate in some countries, although similar categories frequently appear in other theoretical reflections [64,83,126,127]. To increase the value of the framework, we have linked our categorization of policy-makers (strategic, tactical and operational) to internationally familiar management concepts [126,127].

## What are the directions for future research?

By introducing the BCB in the field, we aim to stimulate local policy-makers and those who support them (*e.g.*, researchers) to think about the organizational behaviors that are relevant to developing and implementing integrated public health policies. We want to strengthen the evidence base regarding the reality of policy formulation and implementation, and therefore recommend that researchers apply the BCB in case study designs or narrative inquiries. Such research designs are seen as most appropriate due to their potential to illuminate the dynamic policy process [125,161]. It is our hope that use of the BCB will lead to its further development as a practical and theoretical tool to explore the barriers and facilitators for developing integrated public health policies.

## Summary

This paper has tried to answer some key questions within the context of integrated local public health policies and has introduced a comprehensive framework that can map the various aspects relevant to the development and implementation of such policies. The framework was developed by translating and extending the key assumptions of the ‘Behavior Change Wheel’ (BCW) [27] within a framework called the ‘Behavior Change Ball’ (BCB). Since the BCW and BCB are designed to be applied in different contexts and for different purposes, we propose that both frameworks should co-exist. Throughout our article, we used childhood obesity prevention as an example, since this is a typical wicked problem that requires integrated preventive public health policies. We encourage researchers who are trying to support local policy-makers to apply the framework and report their experiences.

## Competing interests

The authors declare that they have no competing interests.

## Authors’ contributions

AMH carried out the interviews and literature search, and drafted the manuscript. MJ and SK conceived of the study and helped to draft the manuscript. JG, TP, and NdV helped to draft the manuscript. All authors read and approved the final manuscript.
